# Phase-dependent preference of thermosensation and chemosensation during simultaneous presentation assay in *Caenorhabditis elegans*

**DOI:** 10.1186/1471-2202-9-106

**Published:** 2008-11-01

**Authors:** Ryota Adachi, Hiroshi Osada, Ryuzo Shingai

**Affiliations:** 1 Laboratory of Bioscience, Faculty of Engineering, Iwate University, 4-3-5 Ueda, Morioka 020-8551, Japan; 2 Department of Electrical and Electronic Engineering, Faculty of Engineering, Iwate University, 4-3-5 Ueda, Morioka 020-8551, Japan

## Abstract

**Background:**

Multi-sensory integration is necessary for organisms to discriminate different environmental stimuli and thus determine behavior. *Caenorhabditis elegans *has 12 pairs of amphid sensory neurons, which are involved in generating behaviors such as thermotaxis toward cultivation temperature, and chemotaxis toward chemical stimuli. This arrangement of known sensory neurons and measurable behavioral output makes *C. elegans *suitable for addressing questions of multi-sensory integration in the nervous system. Previous studies have suggested that *C. elegans *can process different chemoattractants simultaneously. However, little is known about how these organisms can integrate information from stimuli of different modality, such as thermal and chemical stimuli.

**Results:**

We studied the behavior of a population of *C. elegans *during simultaneous presentation of thermal and chemical stimuli. First, we examined thermotaxis within the radial temperature gradient produced by a feedback-controlled thermoregulator. Separately, we examined chemotaxis toward sodium chloride or isoamyl alcohol. Then, assays for simultaneous presentations of 15°C (colder temperature than 20°C room temperature) and chemoattractant were performed with 15°C-cultivated wild-type worms. Unlike the sum of behavioral indices for each separate behavior, simultaneous presentation resulted in a biased migration to cold regions in the first 10 min of the assay, and sodium chloride-regions in the last 40 min. However, when sodium chloride was replaced with isoamyl alcohol in the simultaneous presentation, the behavioral index was very similar to the sum of separate single presentation indices. We then recorded tracks of single worms and analyzed their behavior. For behavior toward sodium chloride, frequencies of forward and backward movements in simultaneous presentation were significantly different from those in single presentation. Also, migration toward 15°C in simultaneous presentation was faster than that in 15°C-single presentation.

**Conclusion:**

We conclude that worms preferred temperature to chemoattractant at first, but preferred the chemoattractant sodium chloride thereafter. This preference was not seen for isoamyl alcohol presentation. We attribute this phase-dependent preference to the result of integration of thermosensory and chemosensory signals received by distinct sensory neurons.

## Background

While awake, animals detect multiple sensory cues such as chemical and physical stimuli. The variety of sensory information in the environment requires integration of multiple cues within an animal's nervous system. Due to their complexity, neuronal mechanisms that support the multi-sensory integration are difficult to study. The anatomically characterized and compact nervous system of *Caenorhabditis elegans *is composed of only 302 neurons [1], which makes this worm a desirable model organism for studying issues of sensory integration.

*C. elegans *has a sensory organ called an amphid that contains 12 left-right homologous pairs of sensory neurons [2]. More than 100 water-soluble and volatile chemoattractants and repellents are detected by at least one of these sensory neurons, which then direct worms to positive or negative chemotaxis behaviors [3-7]. Sodium chloride is an attractant for *C. elegans*. A previous study revealed that sodium ions are detected mainly by ASEL neuron, with minor contributions by ASER and other neurons, whereas chloride ions are detected mainly by ASER neuron [8]. In contrast, the odorant isoamyl alcohol is detected by AWC neurons [5].

*C. elegans *can also detect physical stimuli, such as tactile and thermal cues [9-11]. AFD neurons are major thermosensors [12] and recently evidence shows that AWC neurons also act as thermosensors in *C. elegans *[13]. AFD neurons play a key role in thermotaxis behavior, which allows worms to navigate toward a preferred temperature, and to track isotherms near that temperature on a spatial temperature gradient. This thermotaxis develops from associative learning between their previous cultivation temperature and feeding state [11,12,14]. Laser ablation technique and the use of mutants has revealed that this thermotaxis behavior has two modes; cryophilic and thermophilic [11,12]. Thermophilic movement is weaker than cryophilic movement and is influenced by new temperature and food state [15]. However, recent behavioral analyses of single worms suggest that the worms do not exhibit thermophilic movement [16,17].

The simultaneous presentation of multiple chemoattractants in *C. elegans *has been studied by examining chemotaxis behavior toward a test attractant within a uniform field of a second attractant [3,5,8,18-22], or toward concentration gradients of two distinct chemosensory stimuli [21-25]. In the latter type of simultaneous presentation experiment, wild-type chemotaxis behavior differed from a sum of the results of single presentation experiments. These studies suggested the presence of informational integration pathways in the *C. elegans *nervous system. Moreover, these worms store temperature and food information during cultivation, so integration of physical and chemical information is thought to occur within their nervous system. Dusenbery *et al*. [26] examined the effect of ambient temperature on chemotaxis behavior toward sodium chloride in wild-type *C. elegans *and showed temperature-dependent alteration in behavior. We extended this analysis to temperature-dependent behavior toward sodium acetate and ammonium chloride, and showed that information from thermosensory AFD neurons is necessary for proper chemotaxis [27].

In the present study, we examined taxis behavior during simultaneous presentation of thermal cue and chemoattractant in *C. elegans*. During the simultaneous presentation assay, wild-type *C. elegans *showed preference to thermal cue in the early phase, and to sodium chloride in the late phase. This phase-dependent preference was not seen in simultaneous presentation of temperature and isoamyl alcohol. We attributed this phase-dependent preference of temperature to the neural integration of thermosensory and chemosensory signals received by distinct sensory neurons.

## Results

### Cryophilic and thermophilic behaviors in the *C. elegans *population assay

**Figure 1:**
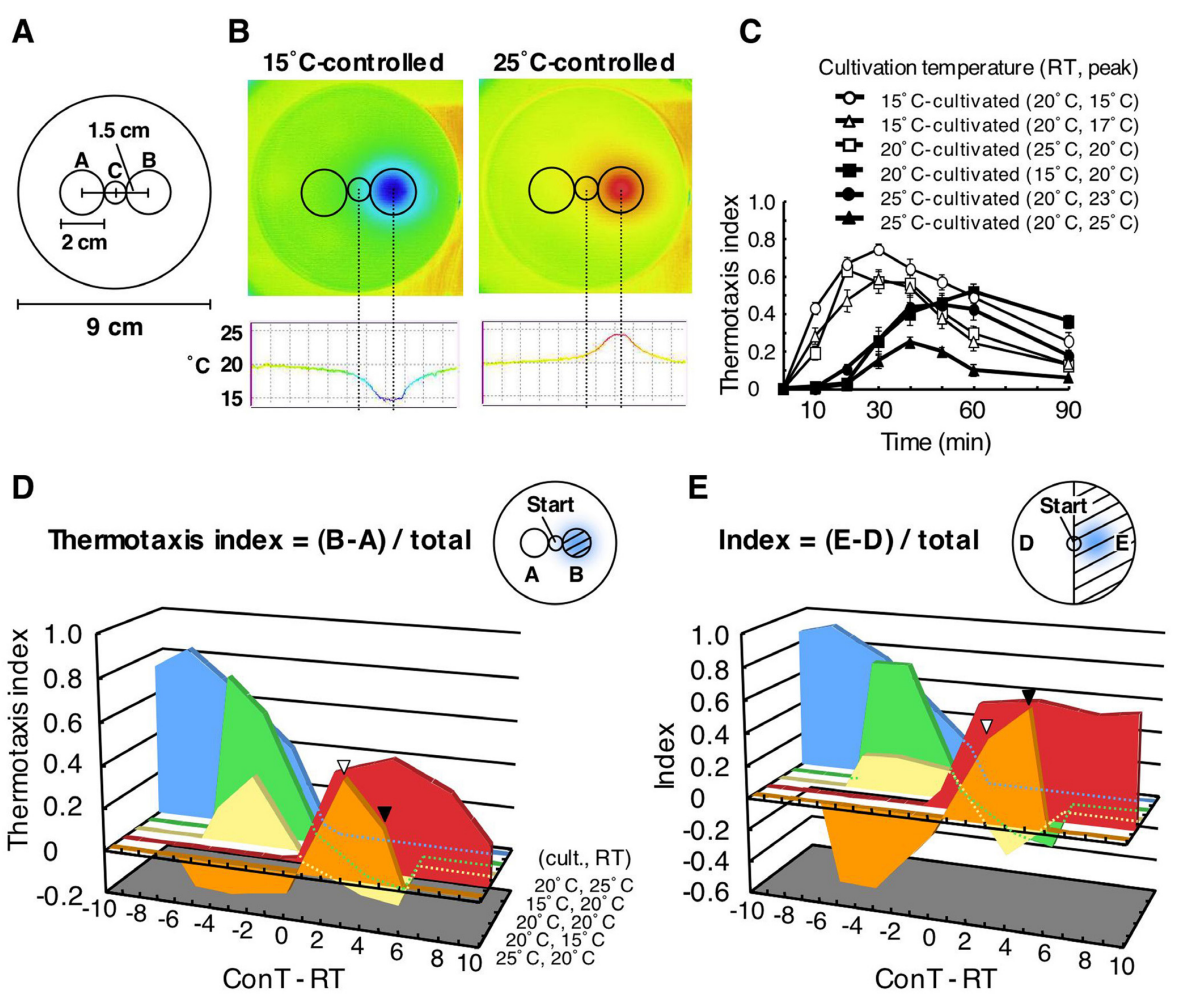
**Thermotaxis behavior**. (A) Configuration of assay plate used in this study. Temperature at the center of region B was controlled and formed a radial temperature gradient. Worms were placed at the center of region C. (B) Temperature gradient of assay plate. (C) Time course of thermotaxis index. RT, peak (e.g., 20°C, 15°C) indicates that room temperature was 20°C and the peak temperature was 15°C. Each data point is presented as the mean ± standard error of the mean (SEM). (D) Relationship between thermotaxis index and temperature gradient. Ordinate indicates the peak values of thermotaxis indices in the worms cultivated at defined temperatures. Steepness of temperature gradient is indicated in the abscissa as a value of controlled temperature at region B (ConT) minus room temperature (RT). (E) Relationship between thermotaxis behavior and temperature gradient. Index (ordinate) indicates the rate of migration toward the side of the plate containing the temperature-controlled region. Abscissa is the same as shown in (D). Arrowheads in D and E indicate indices toward the region of 23°C (white) and toward the region of 25°C (black) in the worms cultivated at 25°C.

To set the conditions for the simultaneous presentation assay, we first confirmed thermotaxis and chemotaxis behaviors in separate assays. Figure 1C shows the time course of worm thermotaxis indices on a defined temperature gradient. Worms that were cultivated below room temperature began to migrate immediately after the start of the assay, and exhibited apparent cryophilic movement (Fig. 1C, unfilled symbols). The thermotaxis index peaked at 20–40 min, and reduced thereafter. By contrast, worms cultivated above room temperature began to migrate 20–30 min after the start of assay, and exhibited thermophilic movement (Fig. 1C, filled symbols). Worms cultivated at 25°C showed a low thermotaxis index (Fig. 1C, filled triangle). The thermotaxis index peaked 40–60 min after the start of assay, and reduced thereafter. Figure 1D shows the relationship between the peak value of the thermotaxis index (ordinate) and temperature gradient (abscissa). The worms cultivated at 25°C exhibited a lower peak thermotaxis index toward the 25°C-region (Fig. 1D, black arrowhead) than toward the 23°C-region (Fig. 1D, white arrowhead). However, the worms migrated well to the 25°C-controlled side of the assay plate (Fig. 1E, black arrowhead) compared with movement to the 23°C-controlled side (Fig. 1E, white arrowhead). These results indicate that worms exhibited thermophilic movement.

### Chemotaxis behavior depends on cultivation temperature

**Figure 2:**
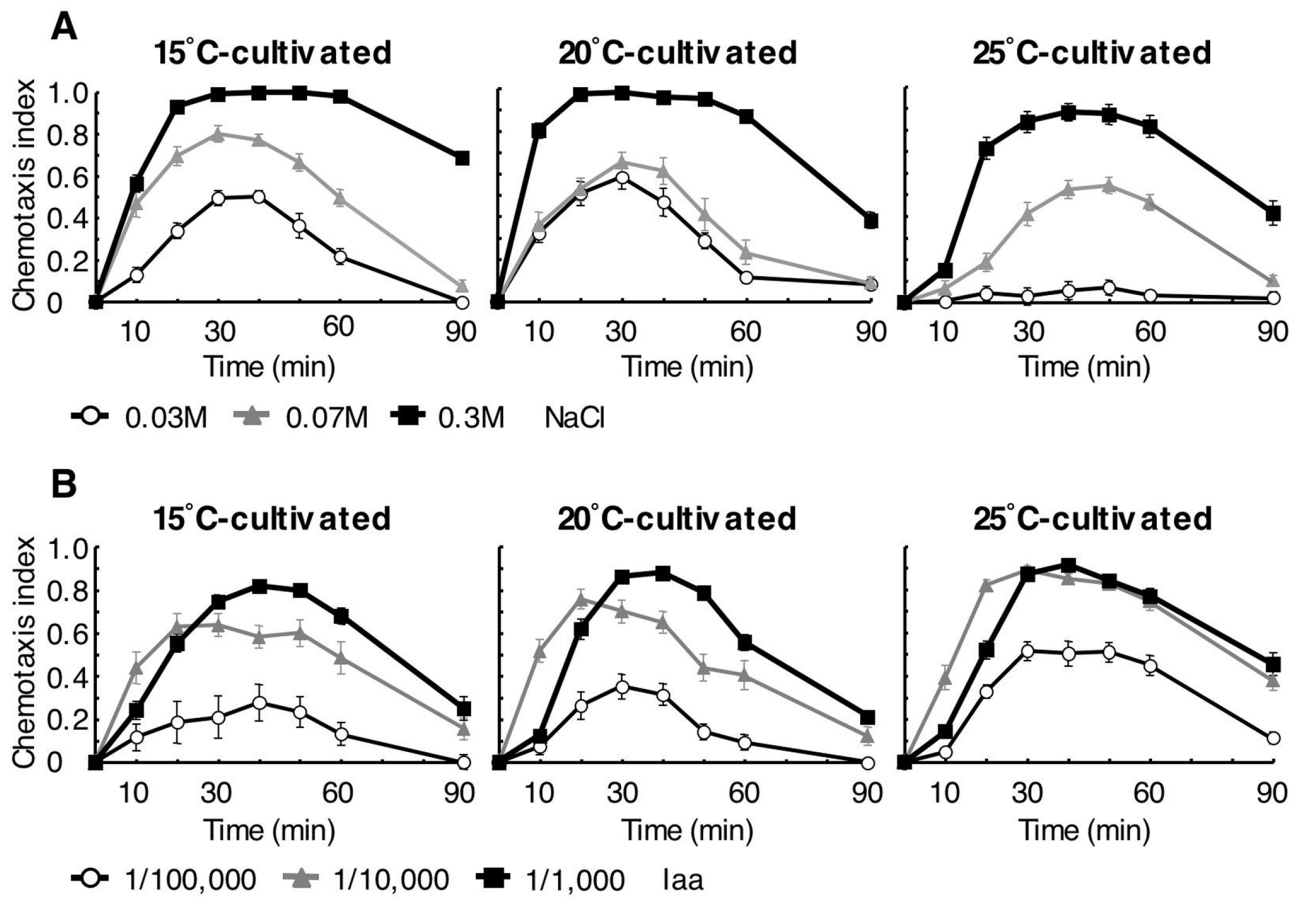
**Chemotaxis behavior**. Sodium chloride or isoamyl alcohol was placed at the center of region A shown in Figure 1A and allowed to form a concentration gradient. (A) Time course of chemotaxis index toward defined concentration of sodium chloride (NaCl). (B) Time course of chemotaxis index toward various dilutions of isoamyl alcohol (Iaa). Worms cultivated at 15°C, 20°C or 25°C were used in each assay. Each data point is presented as the mean ± SEM.

Worms cultivated at 15°C, 20°C or 25°C showed dose-dependent chemotaxis indices at 20°C room temperature (Fig. 2). Concentrations of sodium chloride and isoamyl alcohol designated in Figure 2 were used for the chemotaxis assay. The chemotaxis index peaked 20–40 min after the start of assay, and reduced thereafter. The thermotaxis index followed a time course similar to the chemotaxis index. Sensitivity to sodium chloride tended to decrease as the cultivation temperature increased. Notably, 25°C-cultivated worms did not migrate to 0.03 M sodium chloride (Fig. 2A, right). Unlike the assay with sodium chloride, the chemotaxis index toward isoamyl alcohol tended to increase as the cultivation temperature increased (Fig. 2B). Thus, similar to previously reported temperature-dependent chemotaxis behavior toward sodium acetate and ammonium chloride [27], odor sensing was also affected by cultivation temperature.

### Preference of either temperature or sodium chloride in different phases of simultaneous presentation

To investigate how the integration of multiple sensory signals occurs in *C. elegans*, we studied taxis behavior during simultaneous presentation of spatial temperature and chemical gradients. We used 15°C-cultivated wild-type worms. The region B (Fig. 1A) was maintained at 15°C that is colder than room temperature (20°C). Under this condition, worms exhibited cryophilic movement and migrated toward 15°C. Because cryophilic movement was more evident than thermophilic movement in our assay, we used this condition.

**Figure 3:**
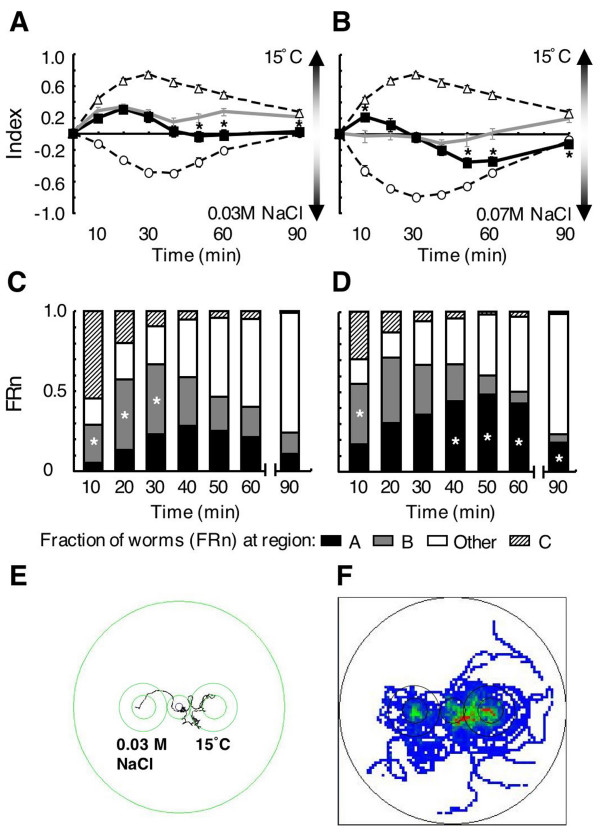
**Simultaneous presentation of 15°C and sodium chloride to 15°C-cultivated worms at 20°C room temperature**. (A and B) Indices of sodium chloride-single presentation (open circle), 15°C-single presentation (open triangle) and simultaneous presentation (closed square). Gray line indicates the sum of the chemotaxis index and the thermotaxis index in single presentations. Each data point is presented as the mean ± SEM. Asterisks indicate significant difference (*p *< 0.05) between simultaneous presentation index and the summed index. In A, 0.03 M sodium chloride was used. In B, 0.07 M sodium chloride was used. (C and D) Fractions of worms (FR_n_) every 10 min during simultaneous presentation assay. FR_other _is fraction in areas other than in the A, B and C regions. Asterisks indicate significant difference (*p *< 0.05) between FR_A _and FR_B_. (E) Track of a single worm under the same conditions as (A). In this example, worms migrated to the region of 15°C first, then to sodium chloride (F) Distribution of tracks of single worms (n = 20). Each track of single worm was recorded for 90 min. High accumulation is indicated by red, and low accumulation is indicated by blue.

When 15°C (cultivation temperature) and 0.03 M sodium chloride were presented simultaneously, worms showed positive indices (i.e., they were attracted more to the 15°C-region than to the sodium chloride-region) for the first 40 min of assay, and they showed nearly zero indices thereafter (Fig. 3A, black solid line). The fraction of worms at a cold region (FR_B_) reduced after the peak at 20 min (Fig. 3C), while the fraction in the sodium chloride-region (FR_A_) peaked at 40 min. We summed thermotaxis indices for 15°C and chemotaxis indices for sodium chloride from separate single presentations (Fig. 3A, gray line). The simultaneous presentation index was almost the same as the summed index for the first 40 min. However, for the last 40 min, the simultaneous presentation index was significantly lower than the summed index (*p *< 0.05).

Interestingly, in the early phase (20 min) of simultaneous presentation of 15°C and 0.07 M sodium chloride (Fig. 3B), worms migrated to a cold region more than the sum of single presentation indices. In the last 40 min of the simultaneous presentation assay, chemotaxis toward sodium chloride was preferred to cryophilic movement, when compared to the summed index (Fig. 3B and 3D).

Single worms were tracked in the simultaneous presentation assay with a computer-driven tracking system. One example is shown in Figure 3E, and all tracks are shown in Figure 3F. Although most of the worms migrated to the 15°C-region or to sodium chloride-region and then moved outside of the attractant regions, some worms migrated again to the other attractant region (sodium chloride or 15°C) (Fig. 3E). These indicate that thermal cue was preferred in the early phase, and chemical cue (sodium chloride) was preferred in the late phase of the simultaneous presentation assay under the condition that the indices in single presentation were mostly equal.

### Preference of temperature or isoamyl alcohol is absent during the simultaneous presentation assay

**Figure 4:**
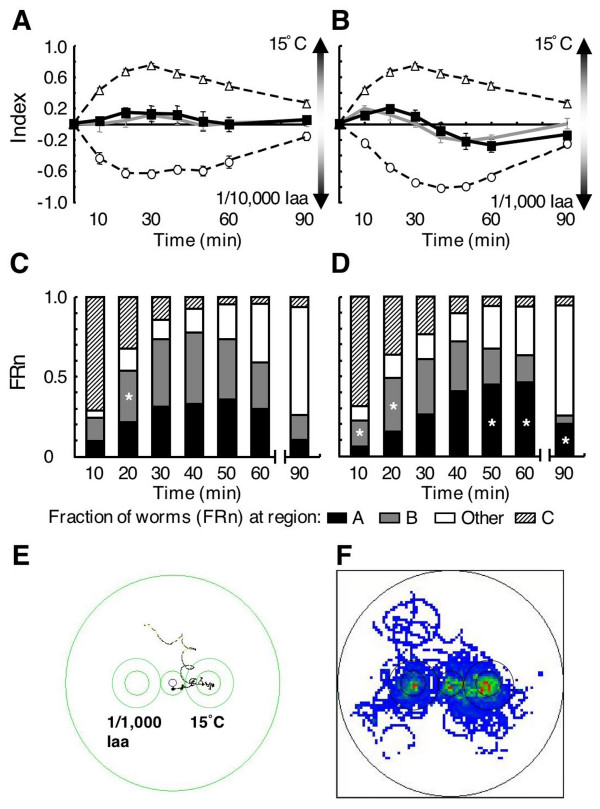
**Simultaneous presentation of 15°C and isoamyl alcohol to 15°C-cultivated worms at 20°C room temperature**. (A and B) Indices of isoamyl alcohol-single presentation (open circle), 15°C-single presentation (open triangle) and simultaneous presentation (closed square). Gray line indicates the sum of the chemotaxis index for isoamyl alcohol and the thermotaxis index for 15°C in single presentations. Each data point is presented as the mean ± SEM. Asterisks indicate significant difference (*p *< 0.05) between simultaneous presentation index (solid line marked with closed square) and the summed index (gray line). Time courses of indices for isoamyl alcohol when 1/10,000 dilution was used (A) and when 1/1,000 dilution was used (B). (C and D) FR_n _every 10 min during simultaneous presentation assay. (E) Track of a single worm under the same conditions as (B). (F) Distribution of tracks of single worms (n = 20). Each track of single worm was recorded for 90 min. High accumulation is indicated by red, and low accumulation is indicated by blue.

When isoamyl alcohol replaced sodium chloride as the chemoattractant, preference to neither the temperature nor the isoamyl alcohol was observed. Under simultaneous presentation of 15°C and 1/10,000 dilution of isoamyl alcohol, the index was nearly zero, and was almost the same as the summed index derived from separate single presentations (Fig. 4A).

When a higher concentration of isoamyl alcohol (1/1,000 dilution) was used, the time course of the simultaneous presentation index (Fig. 4B, black solid line) still resembled that of the summed index (Fig. 4B, gray line). An example of a single worm track is indicated in Figure 4E, and all tracks are indicated in Figure 4F. The worms that migrated to either region did not migrate again to another region, and the rate of migration to both regions was almost equal. These findings indicate that thermal cue and isoamyl alcohol were not distinctly preferred during the simultaneous presentation assay.

### Forward and backward movements of single worms

**Figure 5:**
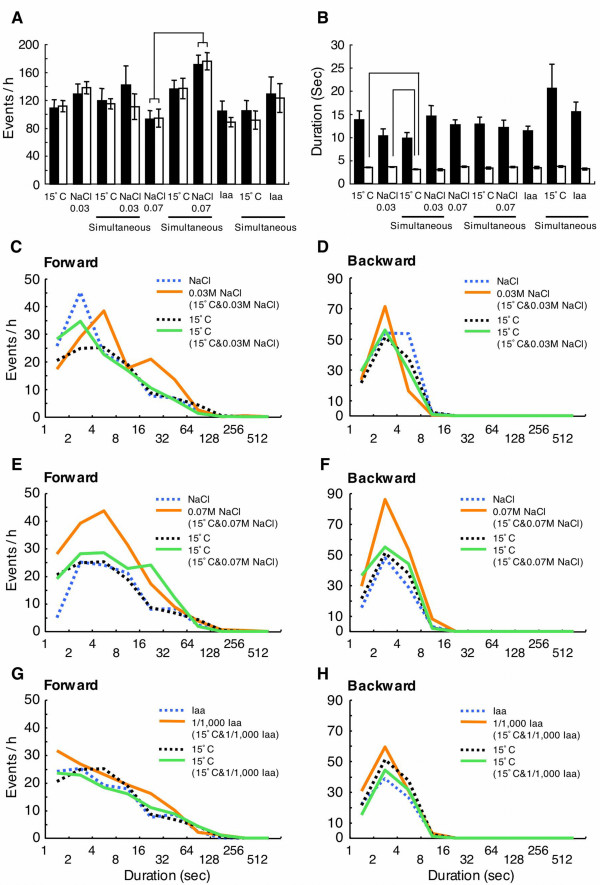
**Analysis of the movement toward an attractant region in single worms**. The frequency (A) and duration (B) of forward and backward movements of single worms were analyzed, and forward (filled bar) and backward (open bar) movements were counted. For single presentations: 15°C, n = 10; 0.03 M sodium chloride, n = 7; 0.07 M sodium chloride, n = 7; isoamyl alcohol, n = 11. For simultaneous presentations: 15°C (with 0.03 M), n = 9; 0.03 M, n = 5; 15°C (with 0.07 M), n = 12; 0.07 M, n = 10; 15°C (with isoamyl alcohol), n = 11; isoamyl alcohol, n = 7. Data are the mean ± SEM. The mean for duration was obtained by averaging the means of durations for individual worms. The data showing significant differences (*p *< 0.01) are linked with a line. (C-H) Distributions of duration of movements. The numbers of movements within a 2^n ^s (n = 1, 2, 3...) interval were summed and expressed as events per hour. The movements of the worms in simultaneous presentation are shown alongside the result from each single presentation.

We analyzed tracks of single worms both in single and simultaneous presentation assays in more detail. In the 90 min assay, we confined our analysis to the movement between the start of the assay and the time when the worm reached either attractant. We did this because, in simultaneous presentation, selecting an attractant might be the result of informational integration in the nervous system, which was done in this period. Worms were divided into two groups, one included worms which migrated to 15°C first, and the other included worms that migrated to the chemoattractant first. The worms in the simultaneous presentation of 15°C and 0.03 M sodium chloride exhibited almost the same frequencies of forward and backward movements as each single presentation assay (Fig. 5A). However, frequencies of forward and backward movements for behavior toward sodium chloride in the simultaneous presentation of 15°C and 0.07 M sodium chloride were significantly higher than those in 0.07 M sodium chloride-single presentation (Fig. 5A). The frequencies of movements in simultaneous presentation of 15°C and isoamyl alcohol were almost the same as those in each single presentation. In contrast, durations of forward movements were not significantly different in the different presentations, except that the duration of backward movement for behavior toward 15°C in simultaneous presentation of 15°C and 0.03 M sodium chloride was significantly shorter than that toward 15°C or 0.03 M sodium chloride in single presentation (Fig. 5B). Therefore, the higher frequency of movements for behavior toward sodium chloride in simultaneous presentation of 15°C and 0.07 M sodium chloride than in each single presentation could be associated with the temperature preference in the early phase of population assay.

Distributions of duration of forward and backward movements are shown in Figure 5C–H. Compared to the case of a separate 0.03 M sodium chloride presentation, movement toward 0.03 M sodium chloride in simultaneous presentation included a decrease in forward movements of short duration (1–4 sec), and an increase in forward movements of long duration (4–8 and 16–64 sec) (Fig. 5C). The peak number of backward movements shifted to a shorter duration in the simultaneous presentation (Fig. 5D). These shifts of the peak number did not change the frequency significantly (Fig. 5A). However, the increment of the number of short backward movements (2–4 sec) could reflect a significant difference between backward movement durations in simultaneous and single presentations (Fig. 5B). In the case of 0.07 M sodium chloride or isoamyl alcohol in simultaneous presentation, both the number of forward movements (Fig. 5E and 5G) and the number of backward movements (Fig. 5F and 5H) increased in wide duration ranges. This increment was evident in the case of 0.07 M sodium chloride and corresponds to high frequency of movements in simultaneous presentation (Fig. 5A). Finally, the number of forward and backward movements in worms that migrated to 15°C in simultaneous presentation was similar to that which occurred in the 15°C-single presentation (Fig. 5C–H).

### Distance and angle from attractant of single worms

**Figure 6:**
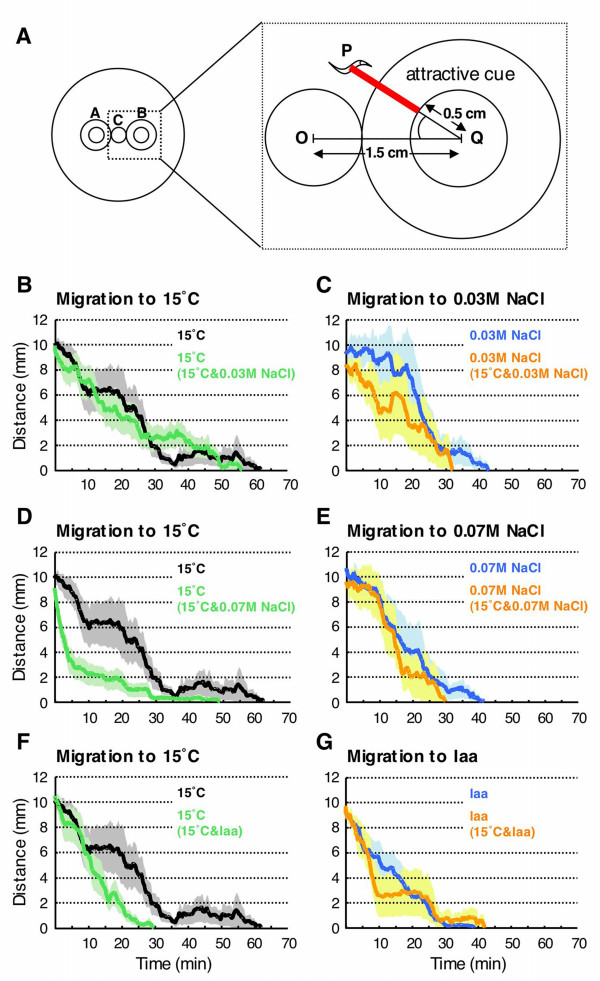
**Analysis of the distance of a worm from an attractant region**. (A) Left: configuration of an assay plate. Right: the distance (red line) and the angle formed by a worm (P), the center of attractant region (Q) and the center of region C (O). The distance and the angle for the attractant in region A are defined similarly. (B-G) The distance of a worm from attractant. The distances in simultaneous presentation of 15°C and 0.03 M sodium chloride (B and C), 15°C and 0.07 M sodium chloride (D and E) and 15°C and isoamyl alcohol (F and G) are shown alongside the results from each single presentation. Each data point is presented as the mean ± SEM. SEM is indicated by grey shading.

To further understand the worm spatial movement, we measured the distance of each worm from the attractant region (Fig. 6). The slope of the distance-time curve was different depending on whether sodium chloride or isoamyl alcohol was used in the assay (Fig. 6B–G). The slope of the distance-time curve of simultaneous presentation assay using 0.03 M sodium chloride was almost same as that of single presentation (Fig. 6B and 6C). Interestingly, migration toward 15°C during simultaneous presentation of 15°C and 0.07 M sodium chloride was evidently faster than during single presentation (Fig. 6D). This may reflect the temperature preference in the early phase of population assay (Fig. 3B). Migration to 15°C during simultaneous presentation of 15°C and isoamyl alcohol was slightly faster than that in single presentation of 15°C (Fig. 6F).

The angle formed between a worm, the center of attractant region, and the center of region C was also measured (Fig. 6A). We confined our analysis to the movement between the start of the assay and the time when the worm reached either attractant. The largest difference between the angles in single and simultaneous presentations was observed for 0.03 M sodium chloride at 10 min. The average angle (± SEM) for migration to 0.03 M sodium chloride in simultaneous presentation at 10 min was 23.7 ± 7.0°, and this value was not different from that for migration to 15°C in simultaneous presentation (9.7 ± 3.1°), but significantly larger (*p *= 0.027) than that for 0.03 M sodium chloride-single presentation (6.6 ± 2.7°). The average angles for migration to 15°C and 0.07 M sodium chloride in simultaneous presentation were 26.1 ± 10.4° and 16.4 ± 6.5°, respectively. These were not significantly different from that for 15°C-single presentation (9.3 ± 1.4°) and that for 0.07 M sodium chloride-single presentation (14.4 ± 2.6°). Other average angles ranged from 9.7 ± 2.3° to 32.1 ± 14.1°, and there was no significant difference among these values.

Furthermore, the average angles at 20 min ranged from 12.7 ± 3.0° to 41.3 ± 11.2°. There was no significant difference among them. The largest difference between the angle at 10 min and that at 20 min was seen for 0.07 M sodium chloride in simultaneous presentation (16.4 ± 6.5° at 10 min and 41.3 ± 11.2° at 20 min), while the angles at 10 min and 20 min for migration to 15°C in this simultaneous presentation were almost the same (26.1 ± 10.4° at 10 min and 29.8 ± 10.3° at 20 min). For 0.07 M sodium chloride-single presentation, the average angle at 20 min was 24.8 ± 7.3° which was smaller value than the case of simultaneous presentation, although the difference was not significant. Therefore, worms approached sodium chloride from a wide angle during the simultaneous presentation assay. These phenomena may correlate with the difference of temperature preference in the early phase of simultaneous presentation of temperature and sodium chloride or isoamyl alcohol.

## Discussion

In this study, we demonstrated multi-sensory integration in the nervous system of wild-type *C. elegans*. Both cryophilic and thermophilic behaviors were observed in the population thermotaxis assay. The simultaneous presentation of temperature and sodium chloride caused worms to exhibit phase-dependent preference for each sensory cue. However, in simultaneous presentation of the temperature and isoamyl alcohol, they did not exhibit phase-dependent preference. Behavioral analysis of single worms indicated that temperature affected the parameters of movement toward sodium chloride (the frequency of movements, and angles to approach the sodium chloride), while 0.07 M sodium chloride affected the slope of the distance-time curve for the migration toward 15°C.

### Thermotaxis behavior of *C. elegans*

We studied thermotaxis behavior on a radial temperature gradient with a population assay. In the single presentation assay, both cryophilic and thermophilic movements were observed, although thermophilic movement was slightly weaker and began about 20 min later than cryophilic movement. Our results suggest that worms cultivated at higher temperatures need some period of time to adjust to a new temperature. Furthermore, 25°C-cultivated worms only weakly migrated to the 25°C-region but migrated well toward the 23°C-region. This is consistent with a previous report that worms cultivated at a higher temperature prefer slightly lower than their cultivation temperature, within linear temperature gradient [28].

We attempted to observe thermophilic behavior of a single worm using a computer-driven tracking system. Although 15°C-cultivated worms migrated to the 15°C-region and tracked isotherms well (n = 24), 25°C-cultivated worms did not migrate to the 23°C-region (n = 20) (data not shown) as reported previously [16]. It is highly possible that there is a difference between thermophilic movement in a single worm and that of a population of worms.

### Phase-dependent preference of attractant in simultaneous presentation

Previous work has shown that *C. elegans *preferentially migrates to either attractant during simultaneous presentation of two distinct chemoattractants [22,24,25]. In the present study, when 15°C-cultivated wild-type worms were placed on an assay plate containing gradients of temperature and sodium chloride, worms preferred to migrate to the 15°C-region in the early phase and to the sodium chloride-region in the late phase. This was different from behavior observed during single presentation assays, and quantitatively different from summed indices of single presentations. Thus, informational integration in *C. elegans *neurons is time-sensitive. Behavioral analyses of single worms (duration and frequency of movement, and angles to approach the attractant) also indicate that the behavior toward sodium chloride is different between single and simultaneous presentations. It is possible that higher frequency of forward and backward movements for behavior toward 0.07 M sodium chloride and the tendency of large angles for sodium chloride at 20 min in simultaneous presentation prevent a fast approach to sodium chloride-region. In contrast, behavior toward the 15°C-region in simultaneous presentation showed a faster approach to this region compared to single presentation. These indicate that the two attractive stimuli quickly disperse worms, and then behavior toward sodium chloride is slowed by frequent forward and backward movements, while behavior toward 15°C is enhanced or less affected by the presence of sodium chloride. This could be a cause of the temperature preference in the early phase of population assay. Our results show that this integration of temperature and sodium chloride information may be different from that of temperature and isoamyl alcohol (See the last section of Discussion).

In the case of the late phase behavior, it would be interesting to suppose that a change of preference from low temperature (15°C) and starvation might have reflected some associative learning in the worms that migrated to the 15°C-region. Mohri *et al*. [15] showed that a decrease in fraction of worms in 17°C-region is proportional to starvation time in the worms pre-cultivated at 17°C. This seems similar to our observation of the decrease in thermotaxis index to the 15°C-region after the peak. However, the interpretation is not straightforward because chemotaxis behavior can also be affected by adaptation [29]. Although, the behavior is complicated in the late phase, the simultaneous presentation index exhibited negative value, that is, sodium chloride was preferred as the result of integration.

### Integration of chemo- and thermo-sensory information

Analyses of behaviors concerning sensory integration have been reported in other invertebrates [30,31]. In the leech nervous system, stimulating identical sensory inputs sometimes elicit crawling and other times swimming [30]. They found a population of single neurons that discriminate before the two behaviors are evident. In *Drosophila*, conditioning with concurrent visual and olfactory cues reduced the threshold for unimodal memory retrieval, and there are crossmodal interactions between olfactory and visual learning [31]. In the present study, worms preferred thermal cue in the early phase of assay. A possible mechanism underlying this behavior is that the memory of cultivation temperature is more effective than attraction of chemical cue for worms. Neurons possibly involved in this behavior are discussed below.

**Figure 7:**
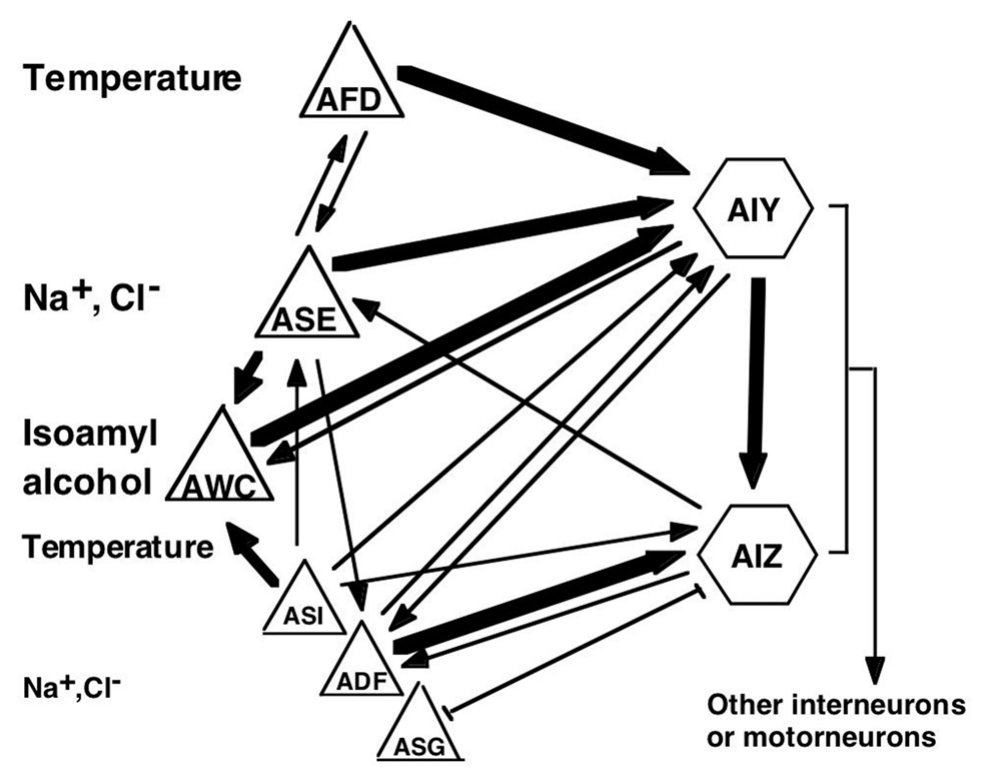
**A partial neuronal network mediating chemotaxis and thermotaxis behavior**. Triangles represent sensory neurons and hexagons represent interneurons. Sodium chloride is detected mainly by ASE, with minor contributions by ADF, ASG and ASI [4]. Thermosensory AFD neurons and AIY and AIZ interneurons play critical roles in thermotaxis behavior [12]. The number of synapses and gap junctions are based on White *et al*. [1]. The arrows represent chemical synapses and the H-shaped bar represents the gap junction. The relative quantity of synaptic connections is represented by line thickness (thin lines: three or fewer synapses; thick line: 16 or more synapses; intermediate line: 4 ~15 synapses).

Recent calcium imaging studies in *C. elegans *revealed that memory of cultivation temperature [32] or thermotactic set-point (Ts), which defines the operating range of AFD thermosensory neurons [14], is stored in the AFD neurons themselves. The *ttx-1 *mutant that is defective in AFD neurons shows abnormal cultivation temperature-dependent chemotaxis [27]. Similarly, AWC neurons also detect temperature, and use same signaling pathway as odor (e.g., isoamyl alcohol) sensing [13]. However, AWC-deleted worms exhibit wild-type-like thermotaxis behavior [13]. Part of the neuronal network involved in thermotaxis and chemotaxis is shown in Figure 7. The ASE neurons detect mainly Na^+ ^and Cl^-^, AWC neurons detect isoamyl alcohol, and AFD and AWC neurons detect temperature. ASE and AFD have mutual synaptic connections, while AWC and AFD have no connections. This difference between ASE and AWC for connection to AFD may affect the difference of temperature preference for sodium chloride and isoamyl alcohol in the early phase of simultaneous presentation. In simultaneous presentation of temperature and sodium chloride, worms might compare the temperature memory in AFD with the sensory information in ASE using their mutual connections, and the combined information is sent to AIY interneurons. Alternatively, the preference of cultivation temperature could be determined in AIY. In simultaneous presentation of temperature and isoamyl alcohol, sensory information received by AFD and AWC would be transmitted to and integrated in AIY interneurons.

HEN-1 is involved in adaptation to sodium chloride and sensory integration of odorant diacetyl and Cu^2+ ^in ASE sensory neurons and AIY interneurons [23]. Because *ttx-3 *mutant animals having defect in AIY interneurons also showed *hen-1 *like phenotype, AIY interneurons are required for integration. In our model, informational integration of temperature and sodium chloride might occur between AFD and ASE as well as in AIY.

By contrast, in the late phase of simultaneous presentation, sodium chloride was preferable to temperature, while isoamyl alcohol was not. This behavior is complex because chemotaxis is affected by adaptation [29], and thermotaxis is affected by associative learning with cultivation temperature and food/starvation [15]. Alternatively, temperature may affect sodium chloride adaptation. Another possibility is involvement of the starvation signal. The starvation signal acts via TAX-6/calcineurin in AIZ interneuron [33]. However, more analysis is necessary to determine whether AIZ neurons are involved in the integration process in the late phase of simultaneous presentation. Further experiments with defined mutants, or lesion techniques such as severing dendrites by femtosecond laser ablation [34], could provide more information about these phenomena.

Why does the worm first prefer cultivation temperature, then prefer sodium chloride? For 15°C-cultivated worms, 15°C may be a metabolically suitable living temperature, even if food does not exist in that environment. Alternatively, associative memory between temperature and food could be stronger than that between chemoattractant and food, in the early phase of simultaneous presentation assay.

## Conclusion

We have demonstrated multi-sensory integration in the nervous system of wild-type *C. elegans*. During simultaneous presentation of temperature and chemoattractant, worms exhibited phase-dependent preference that differed with the kind of chemoattractant. Behavioral analysis of single worms indicated that temperature affected the frequency of forward and backward movements for behavior toward sodium chloride. Also the migration to 15°C-region became faster by the presence of 0.07 M sodium chloride. In the early phase of the assay, the angle for migration to sodium chloride was affected by simultaneously presented temperature. These findings suggest that behavioral preference is the result of integration of thermosensory and chemosensory signals received by distinct sensory neurons, and is time-dependent.

## Methods

### Strains and culture methods

Handling and culturing of *C. elegans *was carried out using established methods [35]. The nematode *C. elegans *Bristol N2 was used as the wild-type strain. This was obtained from the Caenorhabditis Genetics Center.

To obtain synchronously grown young adult hermaphrodites, about 30 gravid adult hermaphrodites were transferred to a nematode growth medium (NGM) plate seeded with *E. Coli*. The NGM plates were kept at 25°C for 3 h to allow worms to lay eggs. Adult worms were then removed from the plates, and the eggs that remained on the NGM plates were incubated at 15°C, 20°C or 25°C until they became young adults. Worms with fully-developed vulva, with no or few eggs in their uteri, and those that exhibited normal pharyngeal pumping were considered young adults and were used for each assay described below. Shortly before the assay, worms were collected in wash buffer (1 mM MgSO_4_, 5 mM KH_2_PO_4_, pH 6.0 adjusted with KOH, 0.005% Tween 20) and washed three times with the same buffer in a 15 ml conical centrifuge tube. A few minutes elapsed while washing the worms, and the temperature during washing was the same as that of cultivation because the buffer was also incubated at each cultivation temperature.

### Thermotaxis assay

A radial temperature gradient on an assay plate was produced by regulating a Peltier device monitored by computer for 1 h. The cupper plate (1 cm in diameter) attached to the Peltier device was contacted with the bottom of a 9-cm diameter assay plate at the center of region B (2 cm in diameter) (Fig. 1A). We set the temperature at region B on the assay plate, regulated by the Peltier device, to 15°C, 17°C, 23°C or 25°C (Fig. 1B). Surface temperature of the assay plate was measured by a digital thermometer (Tweener Thermometer Model-1502A, Netsushin Co., Ltd, Japan) shortly before each experiment, and the temperature gradient on the assay plate was measured by a thermal video system (Super Fine Thermo TVS-8500, Nippon Avionics Co., Ltd, Japan). Regulation of the Peltier device started 1 h before start of an assay. In advance, we confirmed that the surface temperature gradient on the assay plate was stable throughout the assay.

Each assay was started by placing a drop of buffer, containing about 30 washed worms, at the center of the assay plate (region C, 1 cm in diameter). Excess buffer was absorbed, and clumped worms were dispersed with a Kimwipe. Each behavioral experiment was performed for 90 min at 20°C room temperature under the thermoregulation, and a few experiments were performed at 15°C or 25°C room temperature. The room temperature was always monitored with a digital thermometer. The total number of worms in the assay plate was counted, alongside counts for worms in regions A, B and C. The thermotaxis index was calculated as follows: Thermotaxis index = (*N*_B _- *N*_A_)/*N*_total_, where *N*_A_, *N*_B _and *N*_total _refer to the number of worms in the regions A and B, and the total number of worms on the assay plate (Fig. 1B and 1D).

To discuss thermotaxis behavior, another index was introduced. During the thermotaxis assay, the number of worms that migrated to each half of the assay plate was counted (including region B that was thermoregulated). The index was calculated as follows: the index = (*N*_E _- *N*_D_)/*N*_total_, where *N*_D _and *N*_E _are the number of worms on each half of the assay plate (Fig. 1E).

For each data point reported in this assay, we assayed at least 12 plates in at least three independent experiments performed on separate days.

### Chemotaxis assay

Chemotaxis assays were performed with the same format as the thermotaxis assays. The assay plate used in this study was prepared as described previously [27] with some modification. Agar plates 9-cm in diameter were prepared 16–20 h before the assay. A radial concentration gradient of water-soluble chemoattractant was made by placing various concentrations of sodium chloride (7.0 μl) twice onto the center of region A (Fig. 1A), once 15–18 h and once 3 h before running each assay, and kept at 20°C [27]. In the case of a volatile chemoattractant, various dilutions of isoamyl alcohol (1.5 μl) were placed onto the center of region A, and 1.5 μl of 99.5% ethanol (diluent) was also placed onto the other side (region B) of the assay plate shortly before the assay. Although usually sodium azide is used to anaesthetize worms that have migrated into the region of the attractant, we did not use sodium azide in this study.

The chemotaxis index was calculated as follows: Chemotaxis index = (*N*_A _- *N*_B_)/*N*_total_. For each data point reported in this assay, we assayed at least 12 plates in at least three independent experiments performed on separate days.

### Simultaneous presentation assay

The concentration gradient of each chemoattractant was created as described before. These assay plates were set on a Peltier device to obtain a temperature gradient 1 h before each assay. On the assay plate, the peak of the radial temperature gradient was set at the opposite side of the peak of the concentration gradient of chemoattractant (Fig. 1A). Each experiment was performed at the room temperature of 20°C under thermoregulation. The simultaneous presentation index was calculated as (*N*_B _- *N*_A_)/*N*_total_. The fraction of worms FR_n _(n = A, B, C, or other) equals *N*_n_/*N*_total _(n = A, B, C or other) where *N*_other _indicates the number of worms in areas other than in the A, B and C regions. The summed index was defined as summation of the behavioral indices obtained from single presentations temperature (15°C) and chemoattractant (sodium chloride or isoamyl alcohol). Significant differences were detected using the unpaired Student's *t*-test.

### Behavioral analysis of single worms

Locomotion of a single worm on the temperature gradient and/or the concentration gradient of chemoattractant was analyzed by a computer-driven tracking system [36], to which a thermoregulation system was attached. Because the Peltier device was set under an assay plate, LED lights around the assay plate were used as a light source. Configuration of the assay plate was almost the same as described before (see Fig. 1A) except for black paint applied to the bottom of the plate to avoid recording of images beneath the assay plate. Five 15°C-cultivated young adult worms were transferred to wash buffer and washed as in preparation for thermotaxis and chemotaxis assays. Each assay was started by placing a drop of buffer, containing a washed worm, at the center of the assay plate. Excess buffer was absorbed with a Kimwipe. Each experiment was performed for 90 min at 20°C room temperature.

During this 90 min assay, we analyzed forward and backward movements in a period between the start of the assay and the time when the worm reached either attractant. This period ends when the worm arrives at a circumference of a 1-cm diameter circle, which is the same size as the cupper plate attached to the Peltier device in region B. Frequencies of forward and backward movements of an individual worm were obtained by counting the number of events in the period between the beginning of assay and the time when the worm reached the attractant region, and they were converted to the number/h. The mean ± SEM of the frequency was taken for the total worms. Average durations of forward and backward movements of an individual worm were obtained for same the period. Then the mean ± SEM was taken for the average durations of the total worms. Significant differences were detected using the unpaired Student's *t*-test. We measured the distance between worm and a circumference of a 1-cm diameter circle, and the angle formed by a worm (P), the center of attractant region (Q) and the center of region C (O) (see Fig. 6A). Distance and angle were averaged for worms in the region outside the attractant region. We measured angles at 10 and 20 min after the start of assay. Significant difference between the angles under different assay conditions was detected using the unpaired Student's *t*-test, and that between the angles at 10 and 20 min under the same assay condition was detected using the paired Student's *t*-test.

## Authors' contributions

RA carried out all the experiments. HO constructed the thermoregulator. RS conceived the study. RA and RS drafted the manuscript. All authors read and approved the final manuscript.
